# Quantum size effects in TiO_2_ thin films grown by atomic layer deposition

**DOI:** 10.3762/bjnano.5.7

**Published:** 2014-01-22

**Authors:** Massimo Tallarida, Chittaranjan Das, Dieter Schmeisser

**Affiliations:** 1 Applied Physics - Sensors, Brandenburg University of Technology Cottbus–Senftenberg, Konrad-Wachsmann-Allee 17, 03046 Cottbus, Germany

**Keywords:** atomic layer deposition (ALD), charge transfer multiplet, covalency, energy conversion, quantum size effects, titanium dioxide (TiO_2_), water splitting, X-ray absorption spectroscopy (XAS)

## Abstract

We study the atomic layer deposition of TiO_2_ by means of X-ray absorption spectroscopy. The Ti precursor, titanium isopropoxide, was used in combination with H_2_O on Si/SiO_2_ substrates that were heated at 200 °C. The low growth rate (0.15 Å/cycle) and the in situ characterization permitted to follow changes in the electronic structure of TiO_2_ in the sub-nanometer range, which are influenced by quantum size effects. The modified electronic properties may play an important role in charge carrier transport and separation, and increase the efficiency of energy conversion systems.

## Introduction

Titanium dioxide (TiO_2_) is an important material for the photoelectrolysis of water [1] and for many other photocatalytic reactions [2]. Its effective conversion of solar light, although limited by the band gap being too large, has been demonstrated in many systems [3]. Atomic layer deposition (ALD) is a chemical method to grow homogeneous thin films in an atomically controlled mode, which allows for the conformal coating of complex structures with precise thickness and a high degree of purity [4]. The growth of TiO_2_ by ALD is a well-studied process and has been recently reviewed [5]. Charge carrier transport and separation, which strongly depend on interface and surface properties [6–7], are among the most important aspects of energy conversion processes. Therefore the further implementation of efficient photo-electrochemical (PEC) systems is inherently related to the outstanding quality of ALD films, i.e., high purity and homogeneity, and perfect control of thickness in conformal films. It has been shown that very thin films of TiO_2_ may indeed improve PEC performances [8–9].

Recently, TiO_2_ nanoparticles (NPs) with an average diameter of 2 nm showed quantum size effects on unoccupied states [10], which involved the hybridization of Ti 3d and Ti 4s orbitals with O 2p orbitals in covalent bonds. The conformal growth of ALD gives the possibility of having homogeneous films below 2 nm thickness and allows for the investigation of similar quantum size effects in TiO_2_ thin films. In this case interface effects, as those observed in TiO_2_ ALD films grown on SnO_2_:F [11], could also become important. We performed the characterization of ALD films by using mostly X-ray absorption spectroscopy (XAS). Synchrotron radiation (SR) based photoemission spectroscopy (PES) was also used to measure Ti 2p, O 1s and Si 2p core level spectra to determine the films thickness. Further, in order to study the TiO_2_ thin films in the sub-nanometer range, it was decisive to perform the spectroscopic characterization in an in situ ALD system, where the freshly deposited thin films were transported into the measurement chamber without breaking the vacuum [12–13].

The XAS of 3d transition metal (TM) oxides at the O-K and the TM-L_2,3_ edges is a very important tool to determine their electronic and structural properties. Although the detailed interpretation of XAS measurements is very complex and not yet completely achieved, it was shown that rutile, anatase and amorphous TiO_2_ films, as well as quantum-confined TiO_2_ nanostructures exhibit distinct features at both the O-K and the TM-L_2,3_ edges [10,14–15]. Here, we compare our XAS results with previous measurements in order to determine how the degree of covalency of the TiO_2_ thin films can be evaluated and observe that ALD films of TiO_2_ show quantum size effects, which influence their electronic properties.

## Results and Discussion

### Ti-L_2,3_ edge of TiO_2_ thin films

**Figure 1:**
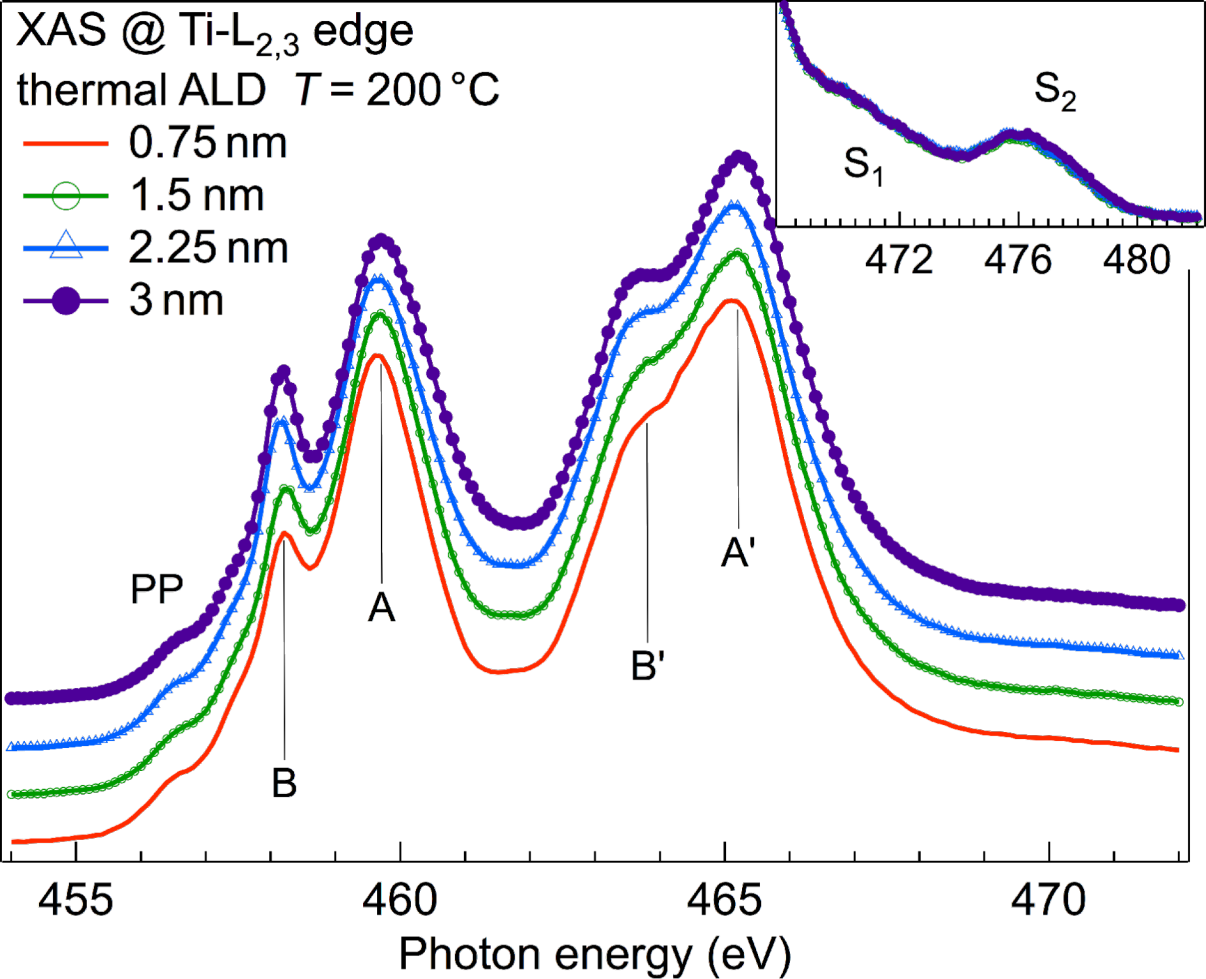
XAS at the Ti-L_2,3_ edge measured for TiO_2_ films with thicknesses of 0.75 nm, 1.5 nm, 2.25 nm and 3 nm. Spectra were normalized to the same intensity at 485 eV and vertically offset.

Thermal ALD of TiO_2_ with titanium(IV) isopropoxide (TTIP) and H_2_O at 200 °C proceeds very slowly [16], with a growth rate of about 0.15 Å/cycle. Indeed, the XAS spectra at the Ti-L_2,3_ edge in Figure 1 show very small changes with increasing number of ALD cycles. Moreover, various spectral features typical of either anatase or rutile TiO_2_ are absent in the XAS spectra of the ALD films [17]. These crystalline phases show a split structure for feature A; a strong and sharp peak B, and distinct pre-preak features PP. Features A′ and B′ are sharper and well separated in the crystalline phases, too. Moreover, in the crystalline phases the satellite features S_1_ and S_2_ are stronger and exhibit a fine structure. Our spectra are, instead, in agreement with those obtained by Kucheyev et al. [14] for amorphous TiO_2_, in which feature A is a broad peak, B is very small, and PP are not well developed. Similar spectra were also observed in very thin TiO_2_ films that were grown by reactive evaporation in oxygen atmosphere at room temperature on SiO_2_ [18], Al_2_O_3_ [19] and MgO [20]. There, XAS spectra at the Ti-L_2,3_ absorption edge were analyzed within the charge-transfer multiplet (CTM) model. Within the CTM theory, the spectral features observed for very thin TiO_2_ films were addressed to a decreased ligand-field at TiO_2_/substrate interfaces, which was found to be increasingly important when moving from the MgO substrate to SiO_2_ [21]. Instead, Krüger used a first-principles multichannel multiple-scattering approach to show that the absence of a split structure in feature A should be addressed to the loss of long-range order on a length scale of 1 nm [22]. Recently, Preda et al. showed for NiO/SiO_2_ that in addition to the lost of long-range order, distortion at the interface induce changes in the XAS spectra [23]. It should be noticed that Ti-L_2,3_ spectra with typical features of anatase and rutile TiO_2_ were obtained with our ALD system when TTIP was used in connection with O_2_-plasma instead of water [17].

### O-K edge of TiO_2_ thin films

**Figure 2:**
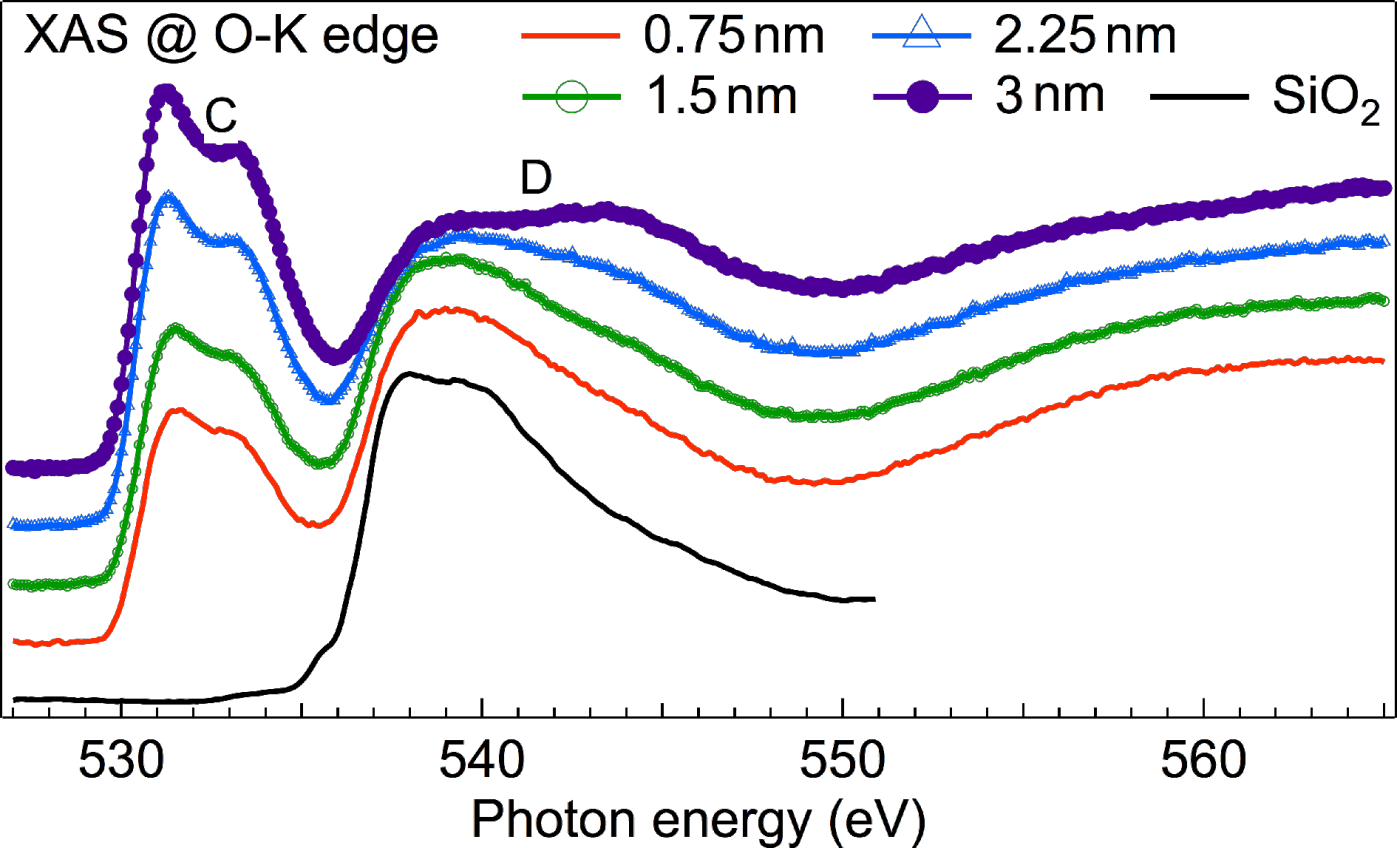
XAS at O-K edge measured for TiO_2_ films with thicknesses of 0.75 nm, 1.5 nm, 2.25 nm and 3 nm. The XAS of native SiO_2_ is also shown as reference. Spectra were normalized to the same intensity at 580 eV and vertically offset.

Differently from the Ti-L_2,3_ spectra, the O-K XAS edge is usually interpreted with the density of unoccupied states of O 2p, as multiplet structures that originate from the overlap of initial and final state wave functions are considered to be negligible [24]. In this case the XAS at O-K edge gives direct information about the hybridization of O 2p with Ti orbitals [24]. The XAS spectra (Figure 2) show mainly two features, namely the double peak C between 530 eV and 535 eV, and the broad feature D extending between 535 eV and 550 eV. The XAS spectra at the O-K edge exhibit evident changes of the shape of the lines with increasing TiO_2_ thickness. In fact, the separation between the two peaks that form feature C, the ratio between feature C and feature D, as well as the shape of the broad feature D vary appreciably after each new deposition.

**Figure 3:**
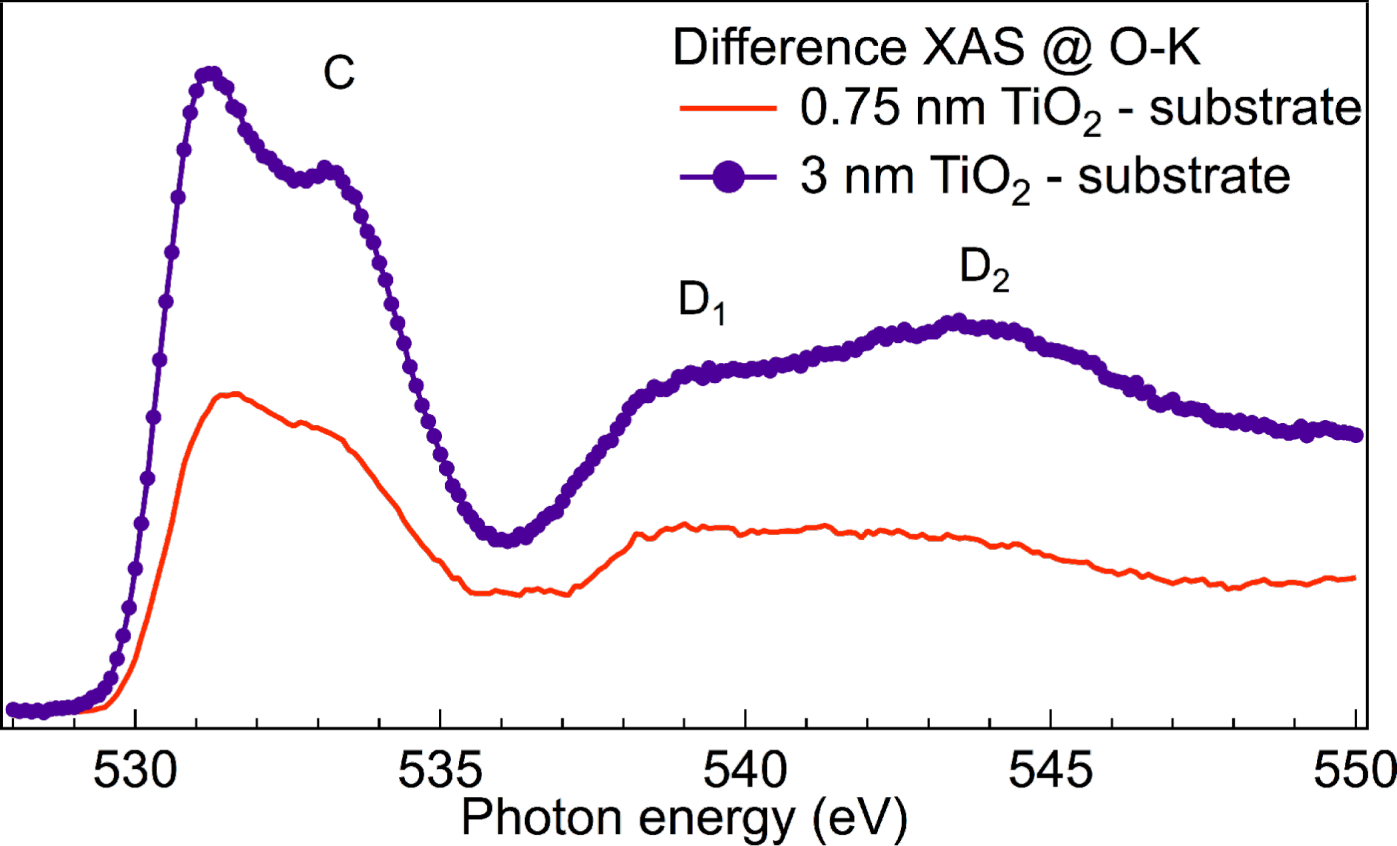
XAS difference spectra. The contribution of SiO_2_ to the XAS at the O-K edge was subtracted from the measured spectra. Feature D of Figure 2 is now described by two features: D_1_ and D_2_.

The shape of feature D is influenced by the presence of SiO_2_ in the substrate, which contributes to the O-K spectra as shown in Figure 2. To remove the contribution of SiO_2_ to the O-K edge of the growing TiO_2_ film, we subtracted the former from each XAS spectrum, as shown for the 0.75 nm and 3 nm films in Figure 3. To this aim we estimated the weight of the SiO_2_ component in the total spectra considering an exponential attenuation. As mean probing depth (MPD) we used the estimation made by Abbate et al. [25], who found that the O-K edges have a MPD of 1.9 nm. For the spectrum of the 0.75 nm film, we subtracted the SiO_2_ substrate spectrum multiplied by 0.68, and for the 3 nm film the same spectrum multiplied by 0.2. Our subtraction procedure is validated by the fact that in a previous experiment [26], in which we deposited HfO_2_ on SiO_2_ and characterized with XAS cycle by cycle, we could observe that the O-K XAS of SiO_2_ does not change during the deposition of HfO_2_.

From the difference spectra the different intensity of peak C and the different shape of feature D, with the latter being formed by two regions, defined as D_1_ and D_2_, become evident. For the 0.75 nm film, the D_1_ contribution is stronger than D_2_ and very broad, while in the 3 nm thick film, the ratio is inversed, D_1_ becomes sharp and D_2_ follows the increase of feature C. Finally, the line shape of the 3 nm film is very similar to that observed in anatase TiO_2_ [10]. Vayssieres et al. found that quantum size effects in TiO_2_ NPs with an average diameter of 2 nm induce a change in the character of the conduction band orbital with a strengthening of the Ti 4s/O 2p hybridization and a simultaneous weakening of the Ti 3d/O 2p hybridization [10]. This was concluded from the observation that the doublet at 530–535 eV (called feature C here) in TiO_2_ NPs was weaker relative to the broad feature in the region 535–550 eV (feature D). The broad region is usually addressed to O 2p states hybridized with Ti 4s and Ti 4p states, and its broadness of about 15 eV is considered to be an indication of strong covalent character of the bonds in TiO_2_ polymorphs, while feature C is usually addressed to Ti 3d/O 2p hybridized states [10]. Although we also observe the increase of feature C and the line shape change of feature D, we additionally notice that the two main structures of feature D (D_1_ and D_2_) in Figure 3 behave differently. This observation establishes an empirical correlation between feature C and the region D_2_ that needs an explanation. Theoretical calculations performed by Wu et al. [27] in the framework of the full multiple scattering theory and the tight-binding linear muffin-tin orbital band-structure method showed the presence of the higher energy tail only for large clusters, which is indicative of a long-range order. On the other hand, the calculation of unoccupied density of states performed by Vayssieres et al. [10] indicate the presence of Ti 4s/O 2p states over a large energy range. Our spectrum of the 0.75 nm film agrees with the calculation of Vayssieres especially in the region of peak D_1_, while peak D_2_ does not have any visible connection to the calculation of small clusters. This could be an indication that peak D_2_ originates not only from Ti 4s/O 2p hybridized states.

**Figure 4:**
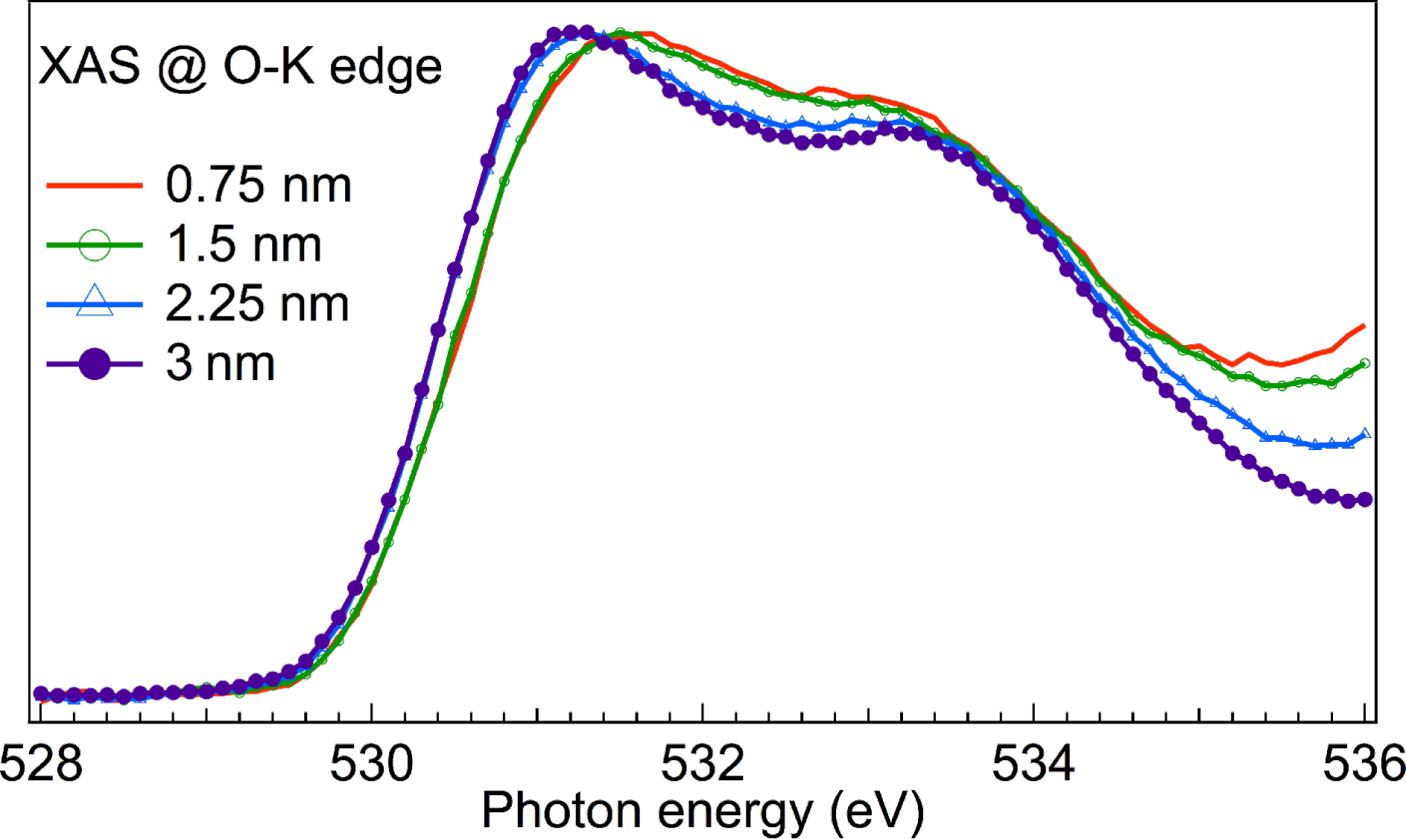
Detailed view of feature C. The spectra were normalized in order to distinguish line-shape changes.

A detailed view of feature C of the four XAS spectra (Figure 4) shows the change of the fine structure in this region when the TiO_2_ film thickness increases from 1.5 nm to 2.25 nm. As mentioned above, the origin of this feature was attributed to hybridized Ti 3d/O 2p states, for which the two peaks appear because the Ti 3d orbital is split into t_2g_ and e_g_ bands by the ligand-field. The shape of 0.75 nm and 1.5 nm TiO_2_ films compares very well with that found by Kronawitter et al. [11] at the interface between TiO_2_ and SnO_2_:F and were attributed to modified electronic properties of TiO_2_. In that case, TiO_2_ was grown by ALD using TiCl_4_ and H_2_O at 150 °C. This shows that the interface formation is similar for these two different ALD-precursors and for different substrates. Kronawitter et al. addressed the changes at the O-K edge to a variation of the ligand-field at the interface, due to structural distortion and to a weaker Ti 3d/O 2p hybridization [11]. We further notice that the decrease of Ti 3d/O 2p hybridization in thinner films pairs up with the increase of Ti 4s/O 2p hybridization, and can be explained by the larger spatial extension of Ti 4s wave functions compared to Ti 3d [10]. These considerations attribute the spectral properties of the TiO_2_ thin films mostly to changes in covalency.

### Covalency in TiO_2_ thin films

Covalency, i.e., the degree of orbital overlap, in TM oxides is a particularly important property for understanding the efficiency of photo-electrodes, as it influences the charge carrier transport mechanism. Covalent materials are desirable because separation and transport of electrons and holes are supported, while the recombination probability of photo-excited carriers is decreased. A change in the covalent contribution to the chemical bond of one material means modified transport properties, and different PEC efficiency [28]. The stronger Ti 4s/O 2p hybridization observed in TiO_2_ thin films causes a larger bandwidth of conduction band states, increases the delocalization of O 2p states, and improves the charge carrier transport. We recently used our TiO_2_ films grown by ALD on Fe_2_O_3_ in order to increase the photoactivated hydrophilic and photocatalytic behavior of Fe oxides. There, it was observed that TiO_2_ thin films and their interface with Fe_2_O_3_ substrates result in an improved charge carrier separation and a decrease of recombination [16] that could be ascribed to the electronic properties of the TiO_2_ thin films.

It is important to understand how changes of the line shape (number, position and intensity of peaks and larger features) in XAS can be related to covalency, and whether these features can be used to estimate variations in PEC efficiency. Determination of the covalent character in a TM–O bond through electronic spectroscopy has been discussed for both XPS [29] and XAS [30]. Although, the O-K edge gives information about covalency by considering the width of broad features and by the intensity of feature C, in the case of Ti-L_2,3_ spectra this interpretation is more difficult and often overlooked because covalency in 3d TM-L_2,3_ spectra goes beyond the (atomic) multiplet model used for the analysis. To include covalency in that model, charge transfer (CT) (from oxygen to TM) is usually adopted in order to include the atomic configurations of the TM with an extra charge density that results from hybridization. For example the purely 3d^0^ atomic multiplet of TiO_2_ is substituted by a combination of configurations, in which 3d^1^L and 3d^2^LL’ CT are partially allowed. However, the atomic nature of the multiplet is still maintained in the calculations [31]. In this way, the influence of covalency in TM-L_2,3_ spectra is mostly related to the presence of peculiar features that are attributed to the charge transfer and not to the relative intensity of the major multiplet features. Instead, these are usually explained with the strength of the ligand-field. The multiplet features are obtained by the transition probabilities to the TM 3d atomic orbital. Due to the strong core–hole effect, the intensity of the various multiplet transitions is weighted by the statistical occupation of the atomic orbital in the initial and final state configurations. Upon changing the ligand-field the energy separation of the d-states (and their statistical occupation) is also changed and the intensity of the main features (obtained from the sum of many multiplets) changes accordingly [31]. In general, a stronger crystal field decreases the intensity of the feature at higher energies and increases the feature at lower energies. Within this model Soriano et al. [21] ascribed the Ti-L_2,3_ XAS of very thin TiO_2_ films to the interaction with the substrate and the decreased ligand-field, which eventually modifies the Ti 3d/O 2p hybridization (defined by the pdσ parameter) because of the modified energy distribution of d-orbitals.

Another way to consider covalency in the framework of atomic multiplets is related to the calculation of Slater integrals. This is usually done by considering isolated ions, by using the one-electron Hartree–Fock method and then rescaled to 80% of their value to account for intra-atomic correlation effects. When a covalent bond is formed, the shape and radial distribution of d-orbitals tend to readjust because of the nephelauxetic effect (typical in TM complexes) [32]. The spatial modification of d-orbitals induces a reduction of the Slater integrals in the covalent bond atoms compared to their ionic values. The reduction of Slater integrals produces a similar effect as that of ligand-field increase, i.e., an intensity increase of the lower energy multiplet transitions and a decrease of the higher energy features [33]. However, the reduction of Slater integrals does not need a structural distortion but only a different charge distribution around covalent bond atoms. From this observation it becomes clear that the same line shape can be simulated within the same theory framework (the charge-transfer multiplet) upon varying two different parameters, i.e., Slater integrals or ligand-field, which have a different physical meaning. While Soriano et al. [21] considered only the changes of ligand-field and pdσ at the TiO_2_ interface with either MgO, Al_2_O_3_ or SiO_2_, attributing the line shape of the Ti-L_2,3_ spectra to structural modifications of TiO_2_, changing the Slater integrals would have induced to attribute the line-shape variations to a changed Ti 3d/O 2p hybridization (and covalency) in very thin films without considering any ligand-field variation, i.e., no structural distortion at the interface. We think that calculations made by varying only the ligand-field parameter could have overestimated its importance, although we agree that at the interface a certain degree of structural distortion should be present. The observed decreased Ti 3d/O 2p (and the increased Ti 4s/O 2p) hybridization at the O-K edge reconciles well with the observed Ti-L_2,3_ spectra in our ALD films when a change of covalency is considered, and calculations with reduced Slater integrals are made maintaining the same or a slightly different ligand-field parameter. This also agrees with the observation that the split of feature A at the Ti-L_2,3_ edge appears in multiple scattering calculations only for large clusters [22], i.e., for systems in which the wavefunction overlap becomes more extended.

## Conclusion

We have shown a detailed characterization of TiO_2_ ALD films with thickness increasing from 0.75 nm to 3 nm by using XAS at both the Ti-L_2,3_ and O-K edges. The use of ALD films permits to have reproducible films with well-defined thickness, and allows a comparison with energy conversion systems, in which thin TiO_2_ films are used to increase efficiency. We deduce that thin TiO_2_ films exhibit peculiar electronic properties, which should be ascribed to quantum size effects. These influence the covalency in TiO_2_ by favouring a delocalization of conduction band states.

## Experimental

Atomic layer deposition (ALD) of TiO_2_ was performed in an ultra-high-vacuum (UHV)-compatible reactor attached to the measurement chamber through a plate valve [12]. The Ti precursor (TTIP) pulse was 4 s, followed by two N_2_ purging pulses, each of 0.5 s, performed just after the Ti precursor and after 5 s. The H_2_O pulse was 0.5 s, followed again by two N_2_ purge pulses. Between the cycles we waited 10 s, in order to have a pressure of 10^−6^ mbar in the ALD chamber before starting the next ALD cycle. The sample was heated inductively to 200 °C. On one Si sample, covered with native SiO_2_, we deposited a TiO_2_ film with increasing thickness, obtained after 50, 100, 150 and 200 ALD cycles. The thickness of the ALD film was 0.75 nm, 1.5 nm, 2.25 nm, and 3 nm, respectively.

Synchrotron radiation was obtained at the U49/2-PGM2 beamline at the BESSY–II synchrotron radiation facility within the Helmholtz-Zentrum Berlin [34]. X-ray photons produced by the U49 undulator were monochromatized by a planar grating monochromator with a resolution of the order of Δ*E*/*E* ≈ 10^−4^. X-ray absorption spectroscopy (XAS) was measured simultaneously while using both total electron yield (TEY) and partial electron yield (PEY). The former was measured through the drain current on the sample, while PEY (and PES) was measured using a PHOIBOS-150 electron analyzer from Specs GmbH, equipped with a 1D delay line detector. The base pressure of the measurement chamber was 5 × 10^−10^ mbar.
